# Data enhanced Hammett-equation: reaction barriers in chemical space†

**DOI:** 10.1039/d0sc04235h

**Published:** 2020-10-02

**Authors:** Marco Bragato, Guido Falk von Rudorff, O. Anatole von Lilienfeld

**Affiliations:** Institute of Physical Chemistry and National Center for Computational Design and Discovery of Novel Materials (MARVEL), Department of Chemistry, University of Basel Klingelbergstrasse 80 CH-4056 Basel Switzerland; Faculty of Physics, University of Vienna Kolingasse 14-16 AT 1090 Vienna Austria anatole.vonlilienfeld@univie.ac.at

## Abstract

It is intriguing how the Hammett equation enables control of chemical reactivity throughout chemical space by separating the effect of substituents from chemical process variables, such as reaction mechanism, solvent, or temperature. We generalize Hammett's original approach to predict potential energies of activation in non aromatic molecular scaffolds with multiple substituents. We use global regression to optimize Hammett parameters *ρ* and *σ* in two experimental datasets (rate constants for benzylbromides reacting with thiols and ammonium salt decomposition), as well as in a synthetic dataset consisting of computational activation energies of ∼2400 S_N_2 reactions, with various nucleophiles and leaving groups (–H, –F, –Cl, –Br) and functional groups (–H, –NO_2_, –CN, –NH_3_, –CH_3_). Individual substituents contribute additively to molecular *σ* with a unique regression term, which quantifies the inductive effect. The position dependence of substituents can be modeled by a distance decaying factor for S_N_2. Use of the Hammett equation as a base-line model for Δ-machine learning models of the activation energy in chemical space results in substantially improved learning curves reaching low prediction errors for small training sets.

## Abstract 2

We generalize Hammett's original approach to predict potential energies of activation in non aromatic molecular scaffolds with multiple substituents.

## Graphical abstract

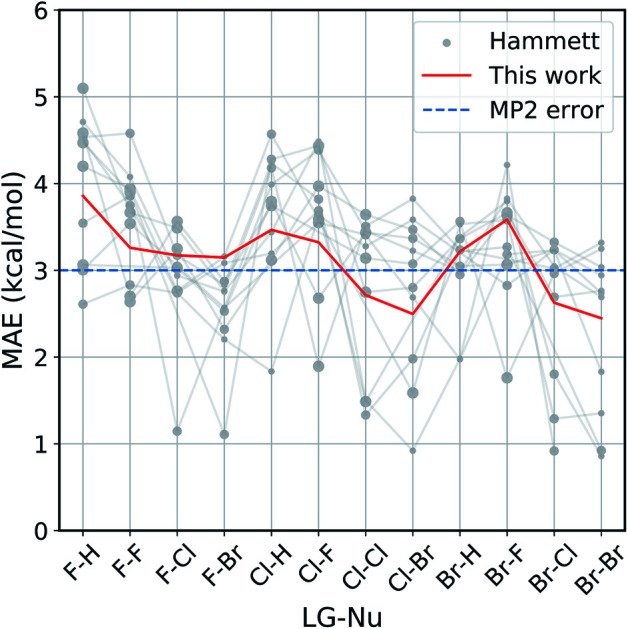

## 1 Introduction

Chemical reactions are difficult to study and model from a theoretical point of view. In 1935, Hammett proposed a quantitative model for free energy differences in benzyl derivatives^1,2^ that assumes that the substituent and reaction effects can be separated by a product ansatz:1
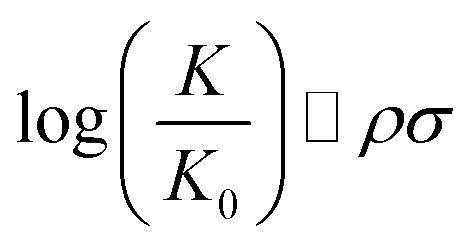
Here, *K* is either the equilibrium or rate constant for a substituted reactant, *K*_0_ refers to the unsubstituted reactant, *ρ* is a constant that depends only on the reaction, taking into account also conditions such as temperature and solvent and *σ* depends only on the type of substituent and its position on the molecule.

This model is compelling since it gives an intuitive concept of electron donating and electron withdrawing effects^3–6^ in the context of free energy differences. The model quickly became quite successful and has been applied to problems ranging from its original purpose, quantifying substituent effects,^3^ to redox potentials,^7^ dipole moments,^8^ orbital energies of metallorganic complexes,^9^ aromaticity,^10–21^ ion stabilization,^22^ mechanicistic investigation,^23,24^ catalyst activity of nanoparticles,^25^ proton–electron coupling in radicals,^26^ molecular conductance,^27^ excited singlet state,^28^ and even toxicities.^29^ More recent approaches have also tried to apply the models to non-benzyl systems.^9,30–32^ It is, however, less satisfying because the linear relationship postulated by Hammett lacks a motivation based on physical effects. Early attempts to explain the theory by electrostatic considerations^33,34^ were successful for special cases only. Nevertheless, Hammett's model has demonstrated remarkable predictive power and accuracy for many cases given the model's simplicity.^3^ Over time the equation has been expanded to also encompass, solvent effect,^35–38^ resonance and field effect,^39^ steric effects,^40–43^ nucleophilicity^44^ and oxidation potential.^45^ These models trade off transferability for accuracy; for this reason, in the majority of applications, the original equation is the one being used.

Hammett's model assumes that substituent effects can indeed be separated from other contributions and are perfectly transferable between environments by virtue of changing *ρ* only, leaving *σ* unchanged. In some sense, Hammett's model therefore captures the part of reality that is directly transferable across chemical environments. Since this assumption is of approximate nature, it is hard to assign unambiguous values of *σ* to functional groups, as they often lack transferability, such that the reference reaction and compound becomes of utmost importance.^46^ Similarly, *ρ* has shown to be hardly transferable and even exhibit an inconsistent temperature dependence.^3^

Interestingly, Hammett parameters can be inferred from experiments: either by OH vibrational frequencies related to the electron density at the point of bonding,^47^ by assessing NMR shifts^48–51^ or quadrupole resonance,^52,53^ by relation to electron binding energies,^54,55^ IR spectroscopy,^56^ electrochemical polarization,^57^ or charge transfer.^58^ Extensive comparison to experiment however, uncovered special cases in which Hammett's model struggles to adequately model reality, partially leading to the introduction of several *σ* values for the same functional group to be used in different molecular environments.^59^ Some limitations subsequently could be surpassed by extending the model, *e.g.* to include concentration dependence.^60^

From a computational perspective, atomic charges were quickly found to correlate with *σ* values for a given functional group,^61–63^ so the few available experimental data points that otherwise would be tedious to extend could be used to calibrate a linear regression while the functional groups were quickly screened by simple charge fitting methods or electron density self-similarity measures.^64^ Still, the resulting *σ* values lack transferability^65^ and computational studies were not successful for reactions involving excited states.^66^ More recently, energy decomposition approaches have been evaluated,^67^ connecting to the idea of electrostatic contributions as a dominating contribution to the validity of Hammett's model.

The use of Hammett's approach as a guide in chemical space to find molecules of desired energy differences has been hampered by three issues: the focus on single substituents, the difficulty to obtain a consistent set of Hammett coefficients^3,68,69^ and the restriction to free energy differences. While multiple substituents have been cautiously explored,^70^ experimental evidence was found that *σ* values of multiple substituents are additive, as long as no resonance is involved.^6,71,72^ In this work, we focus on addressing these three main limitations of Hammett's approach.

## 2 Method

### 2.1 The Hammett equation

The original formulation of the Hammett equation is shown at the beginning of the previous section. Here the only observables are the reaction constants *K* and *K*_0_, so it is not possible to calculate a unique set of {*ρ*} and {*σ*}, as there will always be an arbitrary constant that can be moved between the two. In order to remove this degree of freedom, Hammett proposed the following procedure:^1^ (i) pick a reference reaction i for which *ρ*_i_ = 1, (ii) use it to assign a value of *σ* to the substituents for which there is data for the reference reaction, (iii) use this set {*σ*} to evaluate *ρ*_j_ for another reaction j using a least squares regression, (iv) expand the set {*σ*} using the new *ρ*_j_, (v) repeat steps (iii) and (iv) until each reaction and substituent has a value assigned.

The choice of the reference reaction, as well as the sequence used to expand the set {*σ*}, greatly influences the final result: for a set of *N*_R_ reactions there are up to *N*_R_! possible sets of {*ρ*} and {*σ*}. Overall, with *N*_R_ reactions and *N*_S_ set of substituents there are *N*_R_*N*_S_ different Hammett equations with only *N*_R_ + *N*_S_ parameters to determine. The system is greatly overdetermined, making it easy to overfit the model. Overfitting towards one reference reaction directly reduces transferability of the substituent parameter *σ* across reactions, as Hammett's model reproduces the reference reaction alone. To achieve maximum transferability, a method that is less biased towards one reference reaction is required. In the context of regression, this calls for robust regressors which are less prone to be impacted by observations that do not satisfy the linearity assumptions of Hammett's approach. These observations would constitute outliers for the Hammett regression.

In our model, we use the more robust Theil–Sen regressor,^73^ which evaluates the linear coefficient as the median of the slopes of all lines that pass through each pair of points, and we calculate the entire set of reaction constants {*ρ*} at once. This two additions make the model respectively more robust towards outliers, that could skew the values of the parameters, and remove the dependence on the choice of the reference reaction, which makes the final set of parameters more univoque. The substituent constants {*σ*} are then evaluated by inverting the Hammett equation and averaging the results over all reactions. For numerical reasons, it might be necessary to initially fix one arbitrary reaction constant to 1 to avoid trivial solutions. This is the only source of bias in the model, meaning that the number of possible set of reaction and substituent constant scales only linearly with the number of reactions, and not factorially like in the original model. This procedure allows to affordably identify the best set of parameters. The derivation of the model is explained in details in the ESI.†

For reactants with multiple substituents, *σ* describes the combined effect of all of them. To identify individual contributions, we propose a linear model where the molecular *σ* is given by the sum of single substituent parameters σ̃, obtained by a categorical regression using a dummy encoding. These term depend on the chemical composition of the substituent and on its position on the molecule. In order to separate these two contributions, we modelled each single substituent constant as a product between a term *α*, which depends only the chemical composition, and a distance decaying function (exponential or power law), which encodes the distance of the substituent from the reaction center.

To distinguish the two methods of calculating the substituent constants, *i.e.* by reversing the Hammett equation and by summing single substituents contributions, we named the first one *σ*-Hammett and the latter *α*-Hammett.

Non-linear functions, which can model many body contributions, have also been studied by including three body terms such as the Axilrod–Teller–Muto potential.^74^ This increases the number of parameters needed but allows to include the interactions between substituents.

### 2.2 Machine learning

We trained a Kernel Ridge Regression (KRR) machine to learn the kinetic constant and activation energies for different reactions. Molecules were described with a one-hot encoding representation, which maps every fragment into a fingerprint-like string of zeroes and ones. Our Hammett model was then used as a baseline for Delta Machine Learning^75^ (Δ-ML), where a machine was trained to learn the residuals of the method. This approach can give a faster learning, since the hypersurface of the residuals is usually smoother, thus easier to learn.

These models were programmed in Python using the QML^76^ and scikit-learn^77^ packages. Hyper-parameters were determined with a 5-fold validated grid search, final results obtained with a 15-fold cross validation.

## 3 Results

### 3.1 Experimental analysis

**Fig. 1:**
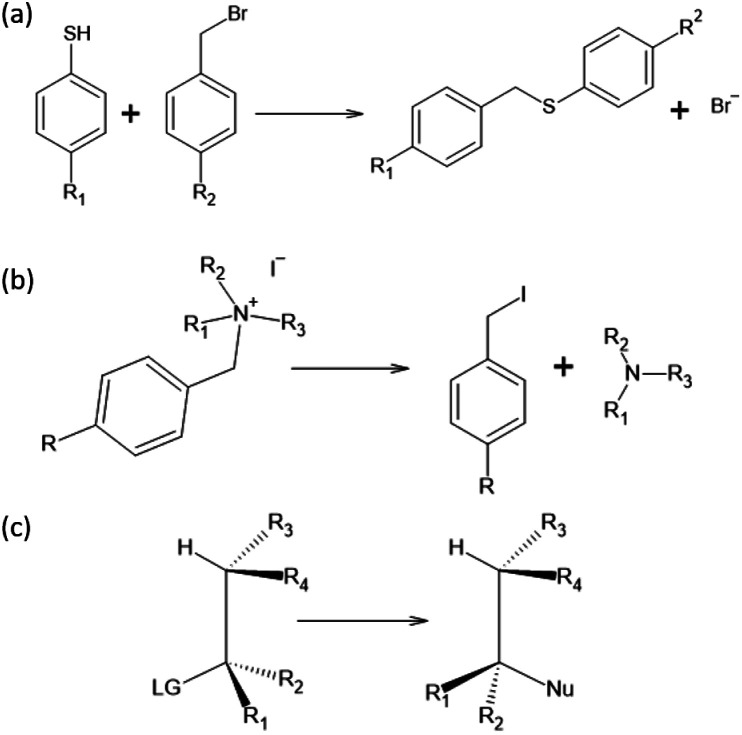
Reactions studied in this paper: (a) nucleophilic reaction between thiophenols and benzylbromides, (b) decomposition of tetra-alkylammonium and (c) S_N_2. Data from reaction (a) and (b) are experimental values while for reaction (c) are computational.

To test the effectiveness of our method, we apply it to two different set of experimental results and compare our predictions with the one from the original Hammett model.^1^ The two reactions are shown in Fig. 1, panels (a) and (b). The first data set^78^ studies the substituent effect on the nucleophilic reactivity between thiophenols and benzylbromides. We use a different *ρ* to describe each reaction with a different thiophenol and a different *σ* for each substituent R_1_ on the benzylbromide. The second data set^79^ reports the rate constants of the decomposition of tetra-alkylammonium salts in solution at different temperatures. According to the original formulation of Hammett, the temperature dependence is included in eqn (1) through the reaction constant, meaning that each temperature is described by a different *ρ*. Each set of substituents on the ammonium salt is then described by a different *σ*.

The kinetic constants have been evaluated through the Hammett equation using three different set of parameters {*ρ*} and {*σ*}: the first one obtained with our model, the second one by applying the original Hammett method, as described in the beginning of the Method section, and the third one using the values of *σ* calculated by Hammett himself in the original paper.^1^ This last method could be used only for the first of the two experimental data set, since the molecules used in the second one where not included in the original paper.

**Fig. 2:**
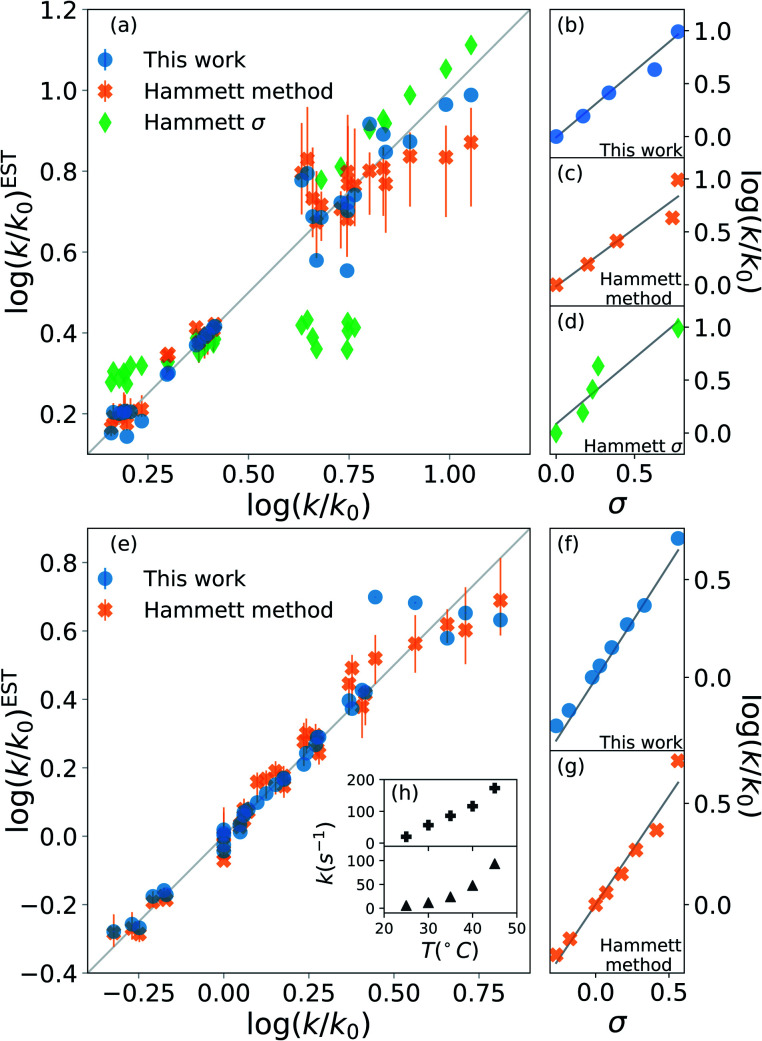
Prediction of kinetic constants on two experimental reaction data set: nucleophilic substitution between benzylbromides and thiols^78^ (top half) and decomposition of ammonium salts (bottom half).^79^ The picture compares results from our model (blue circles), from the original Hammett procedure (orange crosses) and from the tabulated parameters of the original paper (green diamonds).^1^ The correlation plots (a) and (e) show the higher reliability of our method for the prediction of the rate constants when compared to the others. The error bars display the dependence on the reference reaction. The Hammett plots on the right ((b), (c), (d), (f) and (g)) show the increased robustness of our method with respect to outliers and the preservation of the relative ordering of the substituent constants *σ*. The inset (h) reports the temperature dependence of the rate constants for the decomposition of two different ammonium salts, highlighting how the outliers correspond to unphysical behaviour.

The results are shown in Fig. 2. The upper half (subplots (a) to (d)) shows the results on nucleophilic substitution of benzylbromides,^78^ while the bottom half ((e) to (i)) the ones on the ammonium salts decomposition.^79^

The scatter plots (a) and (e) present the correlation between the experimental kinetic constants and the estimated ones. The blue dots are obtained by our model, the orange cross by the original approach^1^ and the green diamond are calculated using the {*σ*} from the original paper.^1^ The error bars show the range of results spanned by changing the reference reaction. For nucleophilic substitution of thiols (upper half), the reference uses the un-substituted thiol, while for the thermal decomposition of ammonium salts (bottom half), the reference is the reaction at 35 °C.

The correlation plots show how our method outperforms the original Hammett method in the vast majority of the case, often by a significant margin; using the original {*σ*} yields very inaccurate results. The error bars demonstrate how important the choice of the reference reaction is: for our method the effect is too small to be visible, while for the original method it can give results that vary by up to 25% for both the first (a) and the second (b) data set. The usage of tabulated sigma removes this dependence but introduces a significant error that can be up to 50%.

The improvement given by our method is in part due to the increased robustness towards outliers. This effect becomes evident from the Hammett plots on the right panels ((b) to (d) and (f) and (e)), which show the linear relationship between substituent constant *σ* and log(*k*/*k*_0_) for each approach. Our method (panels (b) and (f)) gives a better interpolation for the majority of the data. Additionally, the Hammett plots show how the ordering of the different *σ* for different substituents does not depend on the method, meaning that it is still possible to use them as a relative measure of the inductive effect without loss of generality. This comes at the cost of a worse evaluation of the cases that deviate from the linearity.

The tradeoff in accuracy on the outliers is especially evident from the scatter plot (e) for the decomposition of ammonium salts. The original model gives better predictions only for some specific cases, for example when considering the reaction involving a beta-naphtyl thiol. The dependence of the kinetic constant of this last case on the temperature is shown in the top panel of inset (h). The linear behaviour is in contrast with the typical exponential Arrhenius-like that can be observed for any other case in this data set, as presented in the bottom panel of (h) for a *para*-methoxy thiol. This shows that the robustness of the revised Hammett proves useful when dealing with noisy data and can be helpful in identifying unphysical features in the data set.

Overall, Fig. 2 highlights the improvement on the original method given by the application of the Theil–Sen regressor,^73^ which remove the impact of the outliers on the parameters, and by the averaging out of the reference reaction, which significantly reduces the variance for the possible values of the parameters.

### 3.2 Hammett revisited for S_N_2

**Fig. 3:**
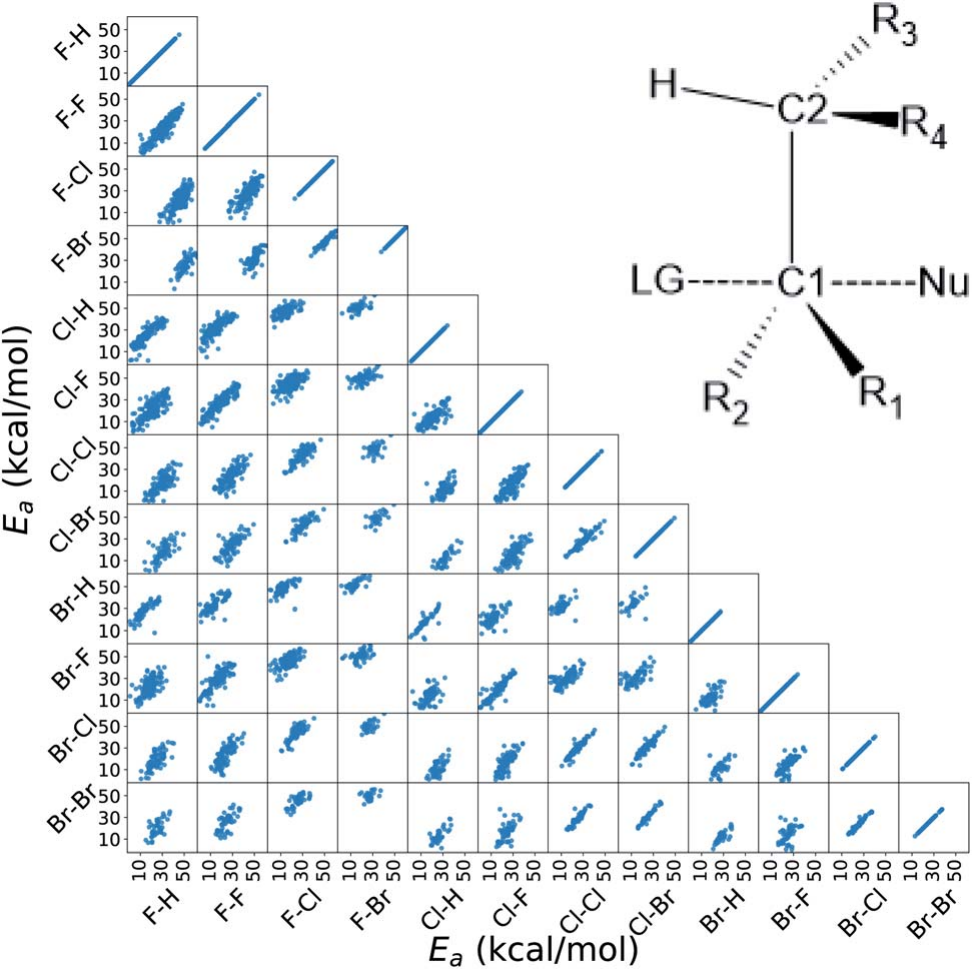
Correlation of the activation energies between the reactions in the data set. The labels indicate the nucleophile-leaving group couple, in this order. The data show a linear trend, which is the underlying assumption for the Hammett model. These activation energies range linearly between 3 kcal mol^−1^ and 40 kcal mol^−1^. The inset in the top right corner shows the general scaffold of the molecules in the data set, where R1 to R4 are the substituents and Nu and LG are the nucleophile and leaving group respectively. The carbon atoms where the substituents are attached are labeled C1 and C2 for the one that undergoes the substitution and the *α* carbon, respectively.

In this work, we extended the Hammett equation to a chemical space that is outside the scope of the original model by working on a computational data set of S_N_2 reactions on small molecules with an ethylene scaffold. The reaction is shown on the bottom of Fig. 1, while the typical transition state is depicted in the top right inset of Fig. 3. These molecules have four sites where substituents can be placed, labelled R_1_ to R_4_, and undergo a nucleophilic substitution of the leaving group LG by the nucleophile Nu. The substituents considered for positions R1 to R4 are –H, –NO_2_, –CN, –NH_3_, –CH_3_, while the leaving groups and nucleophiles are: –H, –F, –Cl, –Br. In total, we consider 12 different S_N_2 reactions, one for each combination of Leaving Group LG and nucleophile Nu, shown on the axis of Fig. 2 and 3. The potential energies have been taken from the QMrxn20 data set.^80^

For this data, we worked with activation energies instead of the kinetic constant. The two quantities are related by the transition state theory, which assumes a quasi-chemical equilibrium between reactants and transition state. Thus, the Hammett equation can be applied to potential energy differences without loss of generality. However, it should be noted that there is an inverse proportionality between kinetic constant and activation energies: a small barrier will be easier to overcome, thus giving a higher kinetic constant, while the opposite is true for a large barrier.

Activation energies for the different reactions correlate linearly with each other, as shown in the lower left part of Fig. 3. Here each scatter plot compares the energy barriers of any two reactions; the nucleophile and leaving group are indicated on the edges, in this order. If Hammett's model was no approximation, all such scatter plots would show perfect linear correlation. We find that the activation energies are strongly correlated meaning that the relative effect of different substituents is the same even across different reactions. Consequently, the ordering of the elements in {*σ*} is unique. The slope of each linear fit expresses the relative susceptibility of the two reactions to the substituents' effect.

**Fig. 4:**
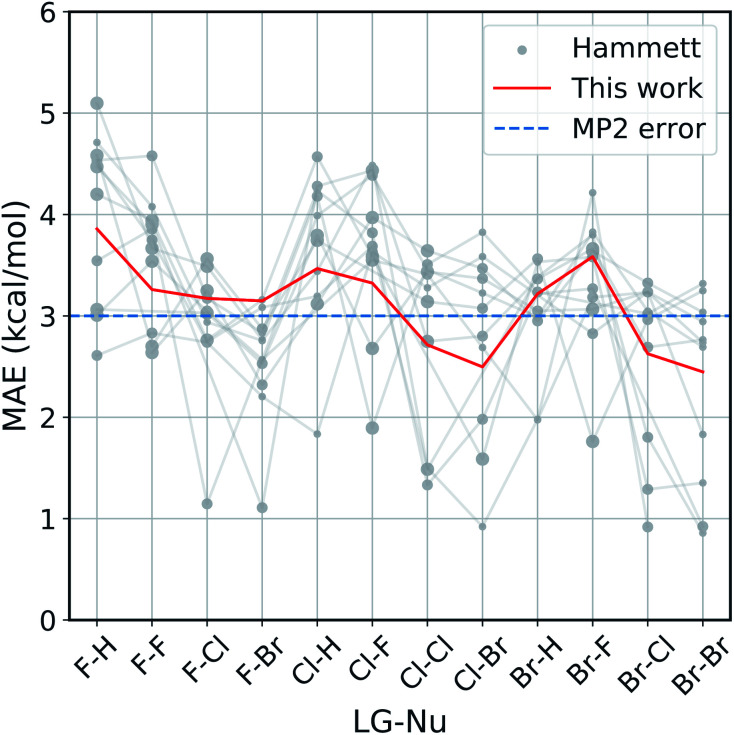
Accuracy of our model with the respect to the original Hammett approach. For each reaction, we show the mean absolute error (MAE) obtained with our model (red line) and with the original Hammett model (gray dots), where each dot represents a different choice for the reference reaction. The size of the dots is proportional to the size of the training set for that data point. The blue dotted line corresponds to the estimated MP2 error.^81,82^

The improvement obtained with our method can be easily seen in Fig. 4. Here we present the Mean Absolute Error (MAE) for the prediction of the activation energy across all the reactions considered. The red line shows the MAE of our model, while the gray dots show the ones of the original model. For each reaction there are eleven dots, one for every different reference reaction in our data set. The thin gray lines connect the results obtained by applying the original Hammett approach to the same reference reaction. The size of each dot is proportional to the number of common set of substituents between the reference reaction and the one being predicted. Finally, the dashed blue line shows the typical error of the MP2 method for nucleophilic reactions,^81,82^ estimated by comparison to W3.2//QCISD.^83,84^

Our method outperforms the classic Hammett approach in the vast majority of the cases. For only a few reactions the original model can give better results but there is no single choice of reference that shows a consistently smaller MAE. Our method averages out the error obtained from the selection bias of the reference and gives a consistent prediction across all reactions, comparable in accuracy to the underlying MP2 method.^82^ Using a higher level of theory could potentially improve the quality of the prediction as long as the different activation energies become more linearly related. It is to be expected though that the dominating linear trend is well-reproduced with MP2 calculations already and that higher level results introduce non-linear corrections to the MP2 energies. In that case, higher level calculations would not improve the Hammett model, as it is only able to capture linear relations and averages out non-linearities. It should be noted that potential energy differences such as activation energies are less susceptible to changes in level of theory.

The original method is highly susceptible to overfitting and numerical noise, as shown by the fact that small errors correspond mostly to medium size dots: few data points (small dots) lead to an unreliable fit, while too many (big dots) can make the model too rigid to be reliably transferable. This is especially evident for the two leftmost reactions (F–H and F–F), were the larger data set are described very poorly by the original model. This can give MAE of up to 5.2 kcal mol^−1^, while our model has an error of 3.8 kcal mol^−1^ at most.

As discussed in the Method section, the original Hammett approach can get up to *N*_R_! different set of parameters, which for the 12 reactions considered here is in the order of 10^8^. The results shown in Fig. 4 are obtained from a regression that considers only the reference reaction and the one for the prediction, so stopping the procedure after only two *ρ* and a subset of *σ* have been assigned. The factorial scaling of the extensive search makes it prohibitively expensive to find the best set of parameters for the original Hammett approach. Since our improvement does not depend on the choice of a particular reference reaction anymore, there is a unique set of model parameters that can be obtained directly, without search.

### 3.3 Decomposition of *σ* for S_N_2

The non-aromatic molecules we considered have four substituents attached to two different carbons atoms: two on the one involved in the reaction, from now on denoted as C1, and two on a carbon atom connected to C1 by a single bond, from now on denoted as C2. The molecular *σ* for each set of substituents depends on all four groups and their position. *Via* categorical regression, described in the ESI,† it is possible to separate the individual contributions σ̃ and express the overall *σ* as a linear combination.

**Fig. 5:**
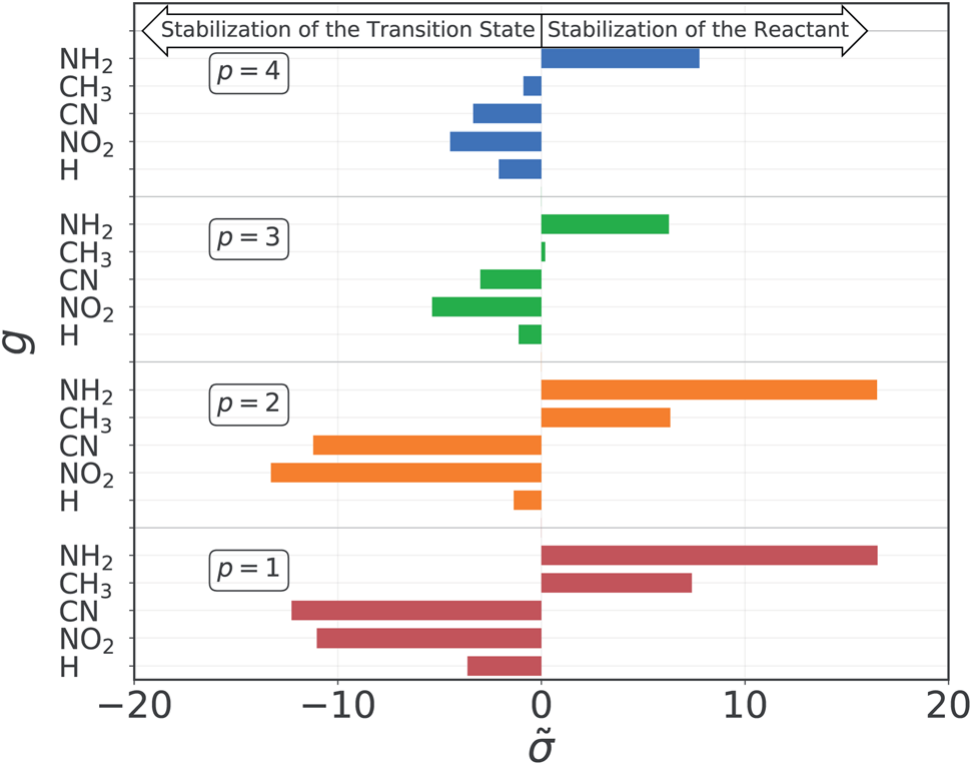
Contribution of each pair of group g and position p to the molecular *σ*, as obtained from the dummy encoding. Positive contributions give larger *σ*, resulting in higher activation energies, while negative contributions lead to a lowered barrier.

The results of the decomposition are reported in Fig. 5. Each horizontal bar corresponds to one single-substituent σ̃ and the colors are used to distinguish the four positions: red and orange for positions 1 and 2, on C1, and green and blue for positions 3 and 4, on C2 (*cf.*Fig. 3). The plot shows that the contributions given by positions 1 and 2 are almost identical. This makes sense chemically, since these two positions are nearly equivalent by symmetry (the molecule is chiral) and thus must have very similar effect on the reactivity of the molecule. The same is true for positions 3 and 4, although their absolute values of σ̃ are much smaller with respect to positions 1 and 2. Again, this follows chemical intuition, as these positions are further away from the reacting centre and their effect is dampened. These two properties of σ̃ are not imposed at any point during the procedure, but they emerge by themselves.

The sign of the single substituent constants can be interpreted in the following way: if the reaction constant *ρ* is positive, a substituent with a negative substituent constant *σ* will give a lower activation energy than the reference substituent, and *vice versa* for positive *σ*. In our case, *ρ* > 0 for all reactions, so it is possible to correlate the single substituent constants with the inductive effect. The electron withdrawing power of the groups considered goes as–NO_2_ > –CN > –H > –CH_3_ > –NH_2_

Groups with negative values are electron withdrawing, while those with positive values are electron donating. This again make sense chemically since the transition state of an S_N_2 reaction is known to be negatively charged, and benefits more from a substituent that can remove electron density from the reacting centre. The discrepancy in the sign with respect to textbook values of *σ* emerges from the fact that here we are considering reaction barriers rather than kinetic constants, and the two properties are inversely related. The correlation with the inductive effect, as well as the magnitude of the substituents' effect depending on the position, is not imposed by the model but shows up naturally during the procedure.

Although the single substituents constant obtained by the categorical regression depend on both their position and chemical composition at the same time, the results of this model indicate that these two can be further separated. We expressed the position dependence as the spatial separation from the reaction center, using a distance decaying function – we tested an exponential and power law one – that scales the electron withdrawing/donating effect of the substituent. The latter is given by a constant which depends only the chemical composition.

The effects of interactions between different substituent on the molecular substituent constant can be modelled by a three-body term, as the Axilrod–Teller–Muto potential.

**Fig. 6:**
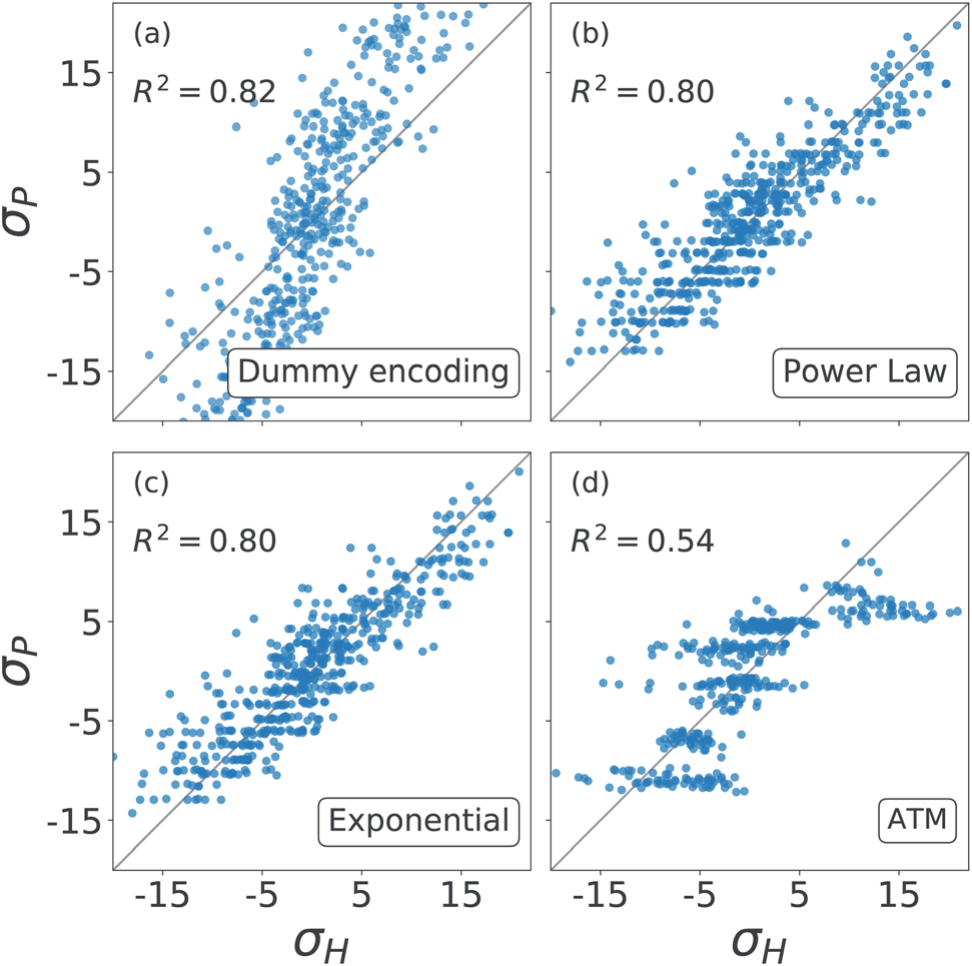
Correlation between the *σ* obtained from the revisited Hammett and the ones obtained from: (a) the dummy encoding, (b) the power law function, (c) the exponential function, (d) the three body Axilrod–Teller–Muto function. Each panel also shows the *R*^2^ of the correlation.

The results from these decompositions of the substituent constants are shown in Fig. 6. Here each scatter plot reports the correlation between the molecular *σ* and the single-substituent ones, obtained with four different prediction methods: (a) categorical regression *via* dummy encoding, (b) power law function, (c) exponential function and (d) Axilrod–Teller–Muto (ATM) function.^74^ Each panel shows the *R*^2^ of the relative fit.

For each of these models, the number of parameters required depends on the number of substituent groups *N*_G_ considered and the number of positions *N*_G_ on the molecular backbone. For our S_N_2 dataset, *N*_G_ = 5 (–H, –NO_2_, –CN, –NH_3_, –CH_3_) and *N*_P_ = 4 (R1, R2, R3, R4) (*cf.*Fig. 3).

The dummy encoding shown in plot (a) requires a total of *N*_P_*N*_G_ parameters, one for each group-position pair, so 20 for this data set. This approach has the great advantage of being independent from the backbone of the molecules, since it is sufficient to label each position and group. Including a new position or group in the data set would increase the number of parameters needed by *N*_G_ (5) and *N*_P_ (4) respectively.

For the exponential function and the power law in panels (b) and (c), the number of parameters required is *N*_G_ + 1, one for each group plus an additional one to regulate the distance decay. For our data set, this means six parameters. In this case, it is necessary to know the geometry of the molecular skeleton, which can be easily obtained. In terms of scalability, adding one more group increases the number of parameters by one, while for a new position it is only necessary to evaluate its distance from the reaction centre. The results obtained by these two functions are very similar, correlate well with the one obtained with the revisited Hammett's algorithm and require less parameters than the categorical regression: in our case we go down from 20 to 6.

The Axilrod–Teller–Muto function shown in panel (d) takes into account the interaction between any two different groups in different positions on the molecule. This requires a total of *N*_G_ + (*N*_G_^2^ + *N*_G_)/2 + 1 parameters: one for each group, one for every unique pair, and an additional one for the distance decay. For our data set, this brings us back to 20, as for the dummy encoding. For the ATM approach it is necessary to know the exact geometries of every molecule in order to calculate the distances and angles between different groups and positions. Extending the data set to a new group increases the parameters' cost by 1 + *N*_G_, *i.e.* 6. Including the interaction between groups and positions removes the simple additivity of single-substituents σ̃ and actually worsens and the prediction.

Overall, Fig. 6 shows that the molecular substituent constants: (i) can be described quite well with only *N*_G_ + 1, *i.e.* 6 parameters, and (ii) show physical additivity. This parametrization allows us to transfer the information gained on one set of substituent to another, making it possible to evaluate the *σ* for a new molecule.

### 3.4 Comparison with machine learning for S_N_2

We compared the performance of our method with a kernel ridge regression machine learning model. We used a one-hot encoding representation, where each molecule is described with fingerprint-like string that depends on the functional groups present. This representation was chosen because it contains no exact structural information, *i.e.* no cartesian coordinates, just like the categorical regression, making the comparison more fair as the two models work with the same information. The machine was trained on both the activation energies and the residuals of the prediction from our revisited Hammett. The latter approach is called delta-machine learning,^75^ and uses as a baseline the predictions obtained from our *α*-Hammett method, described in the Method section and in the ESI,† where the substituent constants are obtained from a linear combination of single substituent contributions that are scaled by a distance decaying function. We choose this method as a baseline because it gives better predictions starting from smaller training set and because its residuals are more consistent, thus easier to learn.

**Fig. 7:**
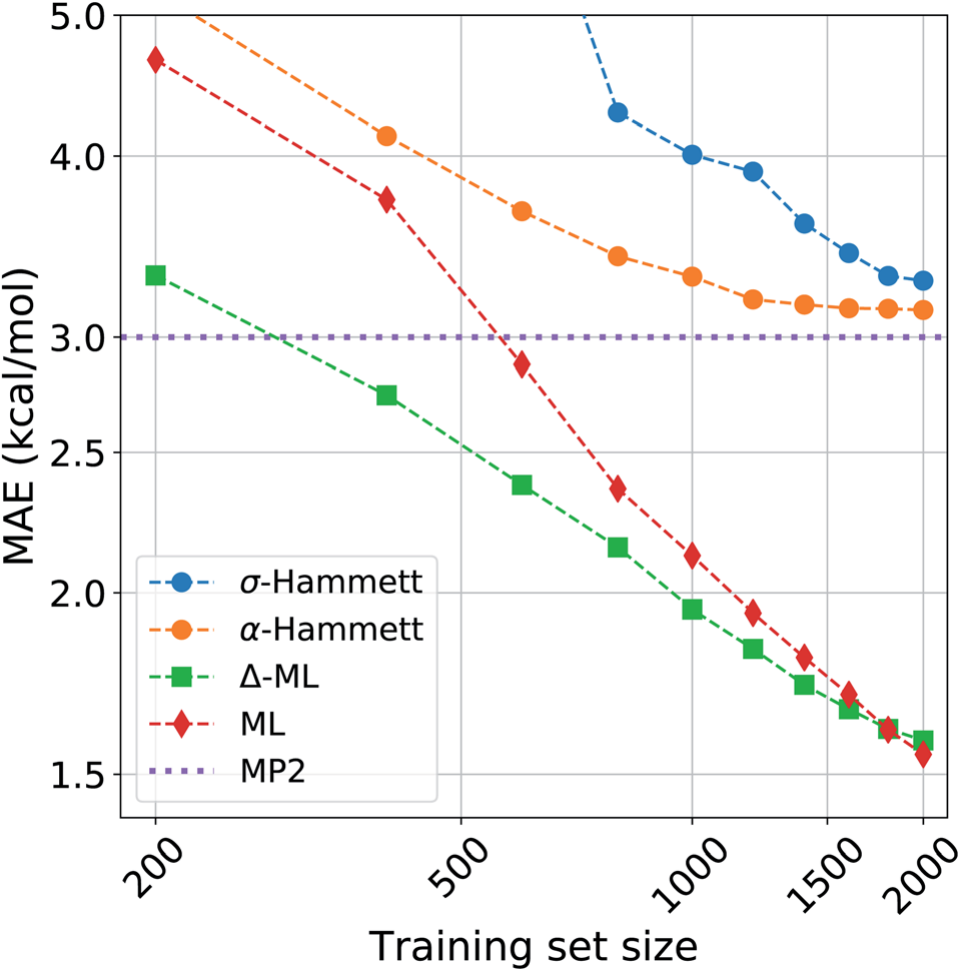
Learning curves for the activation energies with different methods. The circles are obtained by the Hammett model, where the *σ* are calculated globally for the blue line (*σ*-Hammett) and additively for the green line (*α*-Hammett). The diamonds and squares are given by machine learning and Δ-ML respectively, the baseline for the latter is *α*-Hammett.

The comparison of different methods is shown in Fig. 7. Here we report different learning curves, which show how the performance of each method improves as the training set size increases.

For a small training set, only some reactions and set of substituents can be sampled, giving values of *ρ* that are highly influenced by random noise. For the *σ*-Hammett model, this generates a set {*σ*} that poorly reflects the true substituents' effect and gives very high prediction errors. This method shows significant improvement with the increase of the training set size, and using the complete data set recovers the accuracy shown in Fig. 4.

The *α*-Hammett method already gives errors below 5 kcal mol^−1^ for only 400 training points and quickly converges to an accuracy close to the underling level of theory. The flattening out of the learning curve is due to the difficulty of distinguishing similar substituent constants, as also shown by the patter of horizontal lines in Fig. 6.

The ML and Δ-ML methods converge towards the same error, however the latter's learning curve has a significantly lower offset. This means that our method can also be used to speed up the learning of the target property at the cost of a very quick and inexpensive initial treatment of the data. The two learning curves converge at around 1600 data points, where the baseline for the due Δ-ML flattens out. Beyond this point, both methods just learn the MP2 error.

Overall, machine learning consistently outperforms our models in terms of prediction errors. This, however, comes at the cost of a higher complexity of the model, which requires a significantly higher number of parameter and sacrifices chemical interpretability. Kernel ridge regression requires one parameter for each training point, while the Hammett model has only as many parameters as there are reactions and set of substituents. This number is further cut down when considering the *α*-Hammett approach, which for this application makes use of only 18 parameters in total (*cf.* Section 3.3). Each parameter of our model can be understood in terms of inductive effect or susceptibility to it.

The increased flexibility of KRR becomes relevant for deviations from linearity, which the Hammett equation cannot intrinsically handle, as reflected by the flatting out of the learning curves for our models. This occurs when the data in the training set samples the reaction and substituent spaces extensively enough to give stable values for *σ* and *ρ*, and the model cannot give a significant improvement beyond this point.

## 4 Conclusion

We generalized the calculation of Hammett parameters *ρ* and *σ* to account also for potential energy changes due to reactions of non-aromatic molecules with multiple substituents. Our results indicate that substituent effects are largely additive as long as no resonance occurs. For the S_N_2 reaction space, Hammett *σ* values can be explained by chemical composition and distance to the reaction center alone. This connects to the established view regarding the Hammett *σ* values as a measure of the inductive effect, reduces the number of parameters needed by our model and gives each of them a chemical meaning. The decomposition proposed makes it possible to transfer the chemical information gained from one set of substituents to a different one, allowing to estimate the values of *σ* for new molecules. This decomposition in principle would allow future work to extend our approach to resonance cases by assigning *σ* values to pairs (or *n*-tuples) of substituents while retaining the readily interpretable concept of Hammett's model.

Moreover, we present a method to compress quantum chemical reference energies from several reactions into one reliable set of Hammett parameters. This allows to reduce the number of calculations required for real-world applications of Hammett's empirical relationship. Additionally, it reduces the risk of over-fitting towards one specific reaction which we demonstrate to be a significant problem with the original formulation. The overall improvement in robustness over the original method is achieved by using the more robust Theil–Sen regressor for the linear interpolations and by averaging out the influence of the reference reaction.

Our approach builds on the original Hammett equation and it still belongs to the family of linear free energy relationships. The core assumption and main limitation of the model is that a significance variance of the data must be explainable in terms of linear trends. Using higher levels of theory can improved the quality of the prediction as long as this condition is met. Reaction barriers and potential energy differences however, are less susceptible to changes in computational accuracy.

We tested this method on two different experimental data sets and on a computational one and showed systematic and overall improvement in both, prediction quality and reliability. This method also provides an excellent baseline for Δ-ML approaches, effectively forming an valuable stepping stone for dramatically reducing the need for training data obtained from computationally expensive quantum chemistry calculations.

Given modest but sufficient experimental data, and based on the demonstrated improvement of Hammett's empirical formula for potential energies, one can now think of this approach as a more general guideline how to assist in chemical reaction design—without the need of extensive trial-and-error experiments. We rather advocate for diverse data from many different reactions but common molecular skeletons, which then can be combined into one model following our approach. We demonstrated on our data set that this model reaches accuracies similar to quantum chemical calculations. Accordingly, we believe that the promise of Hammett's original idea can now be delivered in order to uncover trends in reaction energetics throughout substantially larger chemical spaces. The code used in our work is freely available.^85^

## Conflicts of interest

There are no conflicts to declare.

## Supplementary Material

SC-011-D0SC04235H-s001
