# Supplementary material for: Data enhanced Hammett-equation: reaction barriers in chemical space
Source: Chem Sci. 2020 Oct 2;11(43):11859–68. doi: 10.1039/d0sc04235h (PMC8163012; doi:10.1039/d0sc04235h)
Supplement: SC-011-D0SC04235H-s001 [file SC-011-D0SC04235H-s001.pdf]

Electronic Supplementary Material (ESI) for Chemical Science.
This journal is © The Royal Society of Chemistry 2020

I. METHOD

1. *The original Hammett procedure*

The Hammett equation was originally intended only for reactions occurring on simple aromatic molecules that have only one substituent on the ring. However, the equation itself contains no assumptions on the structure of the molecule. Due to its linear nature, this equation can be applied to any data set and property P where: (i) the ordering of the substituents with respect to P is mostly stable across all reactions, (ii) the set of values for the property P correlates linearly for any two reactions. The first condition is necessary to have one unique set of substituent constant for every reaction, the second allows to calculate P using only a single multiplicative factor ρ.

2. *Hammett revisited*

The equilibrium constant can be expressed as a function of the free energy difference between product and reactant. The transition state theory extends this formulation to the kinetic constant by assuming a quasi-chemical equilibrium between transition state and reactant, thus using the free energy difference between these two. Both constants can be expressed as:

$$K \propto \exp\left[\frac{-\Delta G}{RT}\right] \tag{1}$$

Thanks to eq 1, we can replace the $\log K$ in the Hammett equation with a free energy difference ΔG or a potential energy difference, since it meets the conditions imposed by the Hammett equation presented above. The logarithm of the kinetic constant can be replace by the activation energy E_a, giving:

$$E_a(s,r) - E_0(r) \simeq \rho(r)\sigma(s) \tag{2}$$

where r is one of the N_R reactions, s one the N_S set of substituents and E_0 is the activation energy for the unsubstituted molecule.

In the following, we describe our approach formally. Moreover, the exact implementation used in this work is freely available[1].

We first evaluate the set of reaction constants $\{\rho\}$. If we compare the activation energies of any two different reactions r_i and r_j which share common set of substituents, we obtain the following system.

$$\begin{aligned} E_a(s,r_i) - E_0(r_i) \simeq \rho(r_i)\sigma(s) \\ E_a(s,r_j) - E_0(r_j) \simeq \rho(r_j)\sigma(s) \end{aligned} \tag{3}$$

Dividing the first equation by the second one gives:

$$E_a(s,r_i) \simeq \frac{\rho(r_i)}{\rho(r_j)}\left[E_a(s,r_j) - E_0(r_j)\right] + E_0(r_i) \tag{4}$$

The ratio between the two reaction constants $\rho(r_i)$ and $\rho(r_j)$ can be obtained via a linear regression of energies, as it is given by the linear slope m of such regression. We made use of a robust regressor [2] to minimize the impact of strong outliers on the final values. This gives a set of $N_R^2 - N_R$ equation of the following form:

$$m\rho(r_j) - \rho(r_i) = 0 \tag{5}$$

These equations can be combined in the linear system:

$$\mathbb{M}\boldsymbol{\rho} = \mathbf{0} \tag{6}$$

Where $\mathbb{M}$ is coefficient matrix with the values for m obtained via equation 5, $\boldsymbol{\rho}$ is the column vector of the N_R reaction constants and $\mathbf{0}$ is a vector of zeroes. Solving equation 6 for $\boldsymbol{\rho}$ will give $\{\rho\}$. For numerical reasons, it might be necessary to initially fix one arbitrary reaction constant to 1 to avoid trivial solutions. This is the only source of bias in the procedure, its effects are discussed below.

Once the $\{\rho\}$ is defined, the substituent constants $\{\sigma\}$ are obtained by averaging the ratio between activation energy and reaction constant across all reactions:

$$\sigma(s) := \frac{1}{R}\sum_{r=1}^{N_R} \frac{E_a(s,r) - E_0(r)}{\rho(r)} \tag{7}$$

We treated each $E_0(r)$ as a model parameter and set it to the median of all the activation energies available for the reaction r. This is done in order to reduce the dependence of the model on only N_R calculations.

This procedure gives a set of substituent constants $\{\sigma\}$ that is much less sensible to reference reaction and the presence of outliers. These $\{\sigma\}$ can then be used to improve the reaction constants $\{\rho\}$ such that they give the best evaluation of activation energies via equation 2. The new values are obtained with a linear regression between $\{\sigma\}$ and $\{E_a\}$. For a specific reaction r_j, the reaction constant $\rho(r_j)$ is given by:

$$\rho(r_j) = \underset{\rho(r_j)}{\arg\min} \sum_{s=1}^{N_S} (\rho(r_j)\,.\,\sigma(s) - E_a(r_j,s))^2 \tag{8}$$

Where the sum runs over all the possible substituents N_s. This procedure can be used to improve the values for $\{E_0\}$ at the same time.

3. *Decomposition of* σ

The substituent constants obtained from eq 7 are molecular properties, which describe the effect of the entire set s of substituents. By denoting each substituted position on the molecule by the index p and each substituent group (e.g. NO_2) by the index g, we highlight the dependency of each σ as $\sigma(s) = \sigma(\{g_p\})$, where by

g_p we indicate the group g to be in position p. If N_P is the total number of positions p on the molecule, and N_G the total number of substituent groups g, the maximum number of set s is $N_G^{N_P}$. However, each molecular σ depends only on N_P terms at most. The overall $\sigma(s)$ can be expressed as a linear combination of these N_P terms:

$$\sigma(s) = \sum_{p=1}^{N_P} \tilde{\sigma}(g_p) \tag{9}$$

The $\tilde{\sigma}(g_p)$ are the single-substituent sigmas. They are independent from one another and can be determined via categorical regression using a dummy encoding. In this, fingerprint-like representation, each molecule in the data set is described by a vector of $N_P N_G$ values, representing all the possible combinations of position and group. All the elements are zeros, except for the ones corresponding to the group-position pairs present in the molecule. These vectors are then stacked into a matrix $\mathbb{A}$ which is then used to solve the linear system

$$\mathbb{A}\tilde{\boldsymbol{\sigma}} = \boldsymbol{\sigma} \tag{10}$$

This type of decomposition reduces the number of parameters needed to describe the substituents from $N_G^{N_P}$ to $N_G N_P$ and allows to predict values of $\sigma(s)$ for set of substituents for which no data is available. However, these $\tilde{\sigma}(g_p)$ still depend on both the position and the group, meaning that the same group will have a different value depending on its position on the molecules. While this is chemically sound, it limits the transferability of the model. To separate the effect of the group g from the one of the position p, we replace the dependence on the latter by distance decaying function that scales the single-substituent effect. This is why the energy difference is modelled after the electronic density. Here an exponential decaying push/pull effect is given by electron withdrawing and electron donating group, respectively.

This can be modelled by the following functions:

$$\sigma(s) = \sigma(\{g_p\}) = \sum_{p=1}^{N_P} \alpha(g) \exp \frac{-d_p}{\tau} \tag{11}$$

$$\sigma(s) = \sigma(\{g_p\}) = \sum_{p=1}^{N_P} \frac{\alpha(g)}{d_p^{\tau}} \tag{12}$$

where $\alpha(g)$ is a parameter which depends only on the group g, regardless of its position on the molecule, d_p is the distance between the position p and the reacting centre on the molecule, and τ is a parameter of the model which regulates the distance decay of the inductive effect. $\alpha(g)$ is determined by a linear regression while the optimal τ can be found by a scan. This approach further cuts down the number of parameters required by the model to describe the substituents from $N_P N_G$ to $N_G + 1$. It requires geometrical information on the backbone of the molecule, which is easily obtainable.

Eq 9, 11 and 12 all neglect the interactions between different group-position pairs. These could be modelled by three body terms such as the Axilrod-Teller-Muto potential form [3]:

$$V_{ijk} = \frac{1 + 3\cos\gamma_i \cos\gamma_j \cos\gamma_k}{r_{ij} r_{jk} r_{ik}} \tag{13}$$

In this case, V is is not a potential, but it keeps the same functional form and includes distances and angles between any two group-position and the reacting centre. This can be used to describe the residuals of the previous fit by including many-body effects. The added flexibility comes at the cost of $(g^2 + g)/2$ additional parameters, one for each possible substituent pair.

4. *Dependence on the reference reaction*

As discussed above, it necessary to initially set on of the reaction constants ρ to 1, in order to avoid trivial solutions. This is the only source of bias in our model and its effect is observed to be limited. For the experimental data sets described in section III.1, the effect of the reference's choice is shown in figure 1. For the computational S_N2 data, we show the influence of the reference choice in figure 1.

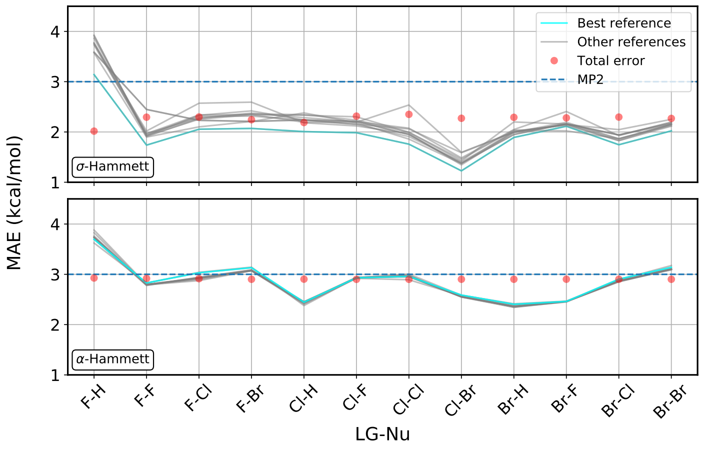

FIG. 1. Influence of the reference reaction on the Mean Absolute Error (MAE) of the prediction of activation energies. Red circles report the overall MAE when the reaction listed on the x-axis is used as a reference. The gray lines, one for each different reference, show the error on the prediction on each specific reaction

The two panels show the Mean Absolute Error (MAE) of the prediction of activation energies. For the top panel,

the substituent constants are obtained from eq 7; we named this method σ-Hammett. In the bottom panel, the substituent constants are obtained from the sum of individual contributions with a power-law distance decay, as calculated from eq 12; we named this method α-Hammett.

Each gray line corresponds to a different choice for the reference reaction, out of the 12 listed on the x-axis, and shows how the MAE changes across the reaction space. In each panel, we highlighted in blue the one that gives the best overall prediction. The red circles show the total error, i.e. across all the 12 reactions, for each reference indicated indicated on the x-axis. These results are compared to the accuracy of the MP2 method, shown by the dashed line.

These plots show how the overall prediction, given by the red circles is only partially affected by the reference bias, especially for the α-Hammett model. Additionally, the gray lines are all very close to each other, meaning that even the description on smaller subset of the data remains mostly consistent regardless of the reference reaction chosen.

The α-Hammett model gives a worse prediction, by about 0.75 kcal/mol on average, but it almost completely negates the effect of the reference bias.

5. *Machine Learning*

The activation energies can also be obtained from Machine Learning. In this work we use Kernel-Ridge Regression, for which the property of interest y of a molecule $\widetilde{X}$ can be predicted as:

$$y(\widetilde{\mathbf{X}}) \simeq \sum_{i}^{N} \alpha_i k(\widetilde{\mathbf{X}}, \mathbf{X}_i) \tag{14}$$

where i runs over all the molecules in the training set, α_i are regression coefficients and $k(\mathbf{X}, \mathbf{X_i})$ is a kernel function. In this work, we used a Laplacian kernel, where each element j, i is given by:

$$k_{j,i} = \exp\left(-\frac{\|\mathbf{A}_j \mathbf{B}_i\|_1}{w}\right) \tag{15}$$

where $\mathbf{A}_j$ and $\mathbf{B}_j$ are representation vectors and w is the kernel width. The regression coefficients α_i can be calculated as:

$$\alpha_i = (\mathbb{K} + \lambda \mathbb{I})^{-1} y \tag{16}$$

where $\lambda > 0$ is a hyperparameter used as a regularizer and $\mathbb{K}$ and $\mathbb{I}$ are the kernel matrix and identity matrix respectively. As representation $\mathbf{X}$ we used the one-hot encoding described in sec. I 3. In this case, the string describes not only the set of substituents, but also the reaction being considered, and for this reason it contains R extra characters, one for each reaction in the data set. This type of machine learning algorithm is used to either predict directly the activation energies or to learn the residuals of the Hammett regression using Delta Machine Learning [4]. The latter works on the assumption that learning the target property from a smoother surface is easier, and thus requires fewer training points to reach high accuracy.

[1] Bragato, M.; von Rudorff, G.; von Lilienfeld, O. A. chemspacelab/Enhanced-Hammett: Enhanced_Hammett. 2020; `https://doi.org/10.5281/zenodo.3952671`.

[2] Theil, H. *Nederl. Akad. Wetensch., Proc.* **1950**, *53*, 386–392 = Indagationes Math. 12, 85–91 (1950).

[3] Axilrod, B. M.; Teller, E. *The Journal of Chemical Physics* **1943**, *11*, 299–300.

[4] Ramakrishnan, R.; Dral, P. O.; Rupp, M.; von Lilienfeld, O. A. *Journal of Chemical Theory and Computation* **2015**, *11*, 2087–2096, PMID: 26574412.
